# Predicting tumour resistance to paclitaxel and carboplatin utilising genome‐wide screening in haploid human embryonic stem cells

**DOI:** 10.1111/cpr.13771

**Published:** 2024-11-10

**Authors:** Jonathan Nissenbaum, Emanuel Segal, Hagit Philip, Rivki Cashman, Tamar Golan‐Lev, Benjamin E. Reubinoff, Adi Turjeman, Ofra Yanuka, Elyad Lezmi, Oded Kopper, Nissim Benvenisty

**Affiliations:** ^1^ NewStem LTD Jerusalem Israel; ^2^ The Azrieli Center for Stem Cells and Genetic Research, Department of Genetics, Silberman Institute of Life Sciences The Hebrew University Jerusalem Israel; ^3^ Hadassah Stem Cell Research Center, Goldyne Savad Institute of Gene Therapy, Department of Obstetrics and Gynecology Hadassah Hebrew University Medical Center Jerusalem Israel; ^4^ The Center for Genomic Technologies The Hebrew University Jerusalem Israel

## Abstract

Taxanes and platinum molecules, specifically paclitaxel and carboplatin, are widely used anticancer drugs that induce cell death and serve as first‐line chemotherapy for various cancer types. Despite the efficient effect of both drugs on cancer cell proliferation, many tumours have innate resistance against paclitaxel and carboplatin, which leads to inefficient treatment and poor survival rates. Haploid human embryonic stem cells (hESCs) are a novel and robust platform for genetic screening. To gain a comprehensive view of genes that affect or regulate paclitaxel and carboplatin resistance, genome‐wide loss‐of‐function screens in haploid hESCs were performed. Both paclitaxel and carboplatin screens have yielded selected plausible gene lists and pathways relevant to resistance prediction. The effects of mutations in selected genes on the resistance to the drugs were demonstrated. Based on the results, an algorithm that can predict resistance to paclitaxel or carboplatin was developed. Applying the algorithm to the DNA mutation profile of patients' tumours enabled the separation of sensitive versus resistant patients, thus, providing a prediction tool. As the anticancer drugs arsenal can offer alternatives in case of resistance to either paclitaxel or carboplatin, an early prediction can provide a significant advantage and should improve treatment. The algorithm assists this unmet need and helps predict whether a patient will respond to the treatment and may have an immediate clinically actionable application.

## Graphical abstract

To gain a comprehensive view of genes that affect the anticancer drugs paclitaxel and carboplatin resistance, genome‐wide loss‐of‐function screens in haploid hESCs were performed. The screens yielded selected plausible gene lists and pathways relevant to drug resistance, and an algorithm that can predict resistance to paclitaxel or carboplatin was developed.

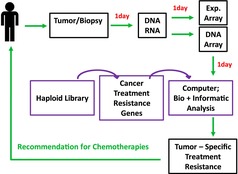

## 1 INTRODUCTION

Induction of cell death is a pivotal mechanism in fighting cancer. Various methods and therapies such as irradiation, inhibitors of checkpoint control, and global cytotoxicity are all useful options that aim to eliminate tumours. Taxanes and platinum molecules are widely used anticancer drugs which induce cell death and serve as first‐line chemotherapy for various cancer types.^1^, ^2^, ^3^ Among those molecules, paclitaxel and carboplatin are highly prevalent in the clinic.^4^, ^5^, ^6^, ^7^ Their mode of action is different though: paclitaxel interferes with tubulin composition (as a microtubule stabiliser) and spindle assembly,^8^ while carboplatin covalently binds to DNA, causing the formation of DNA cross‐links and inhibition of DNA replication.^9^ Despite the efficient inhibition of cancer cell proliferation by both paclitaxel and carboplatin, tumours may have innate resistance against the drugs, which leads to inefficient treatment and poor survival rates.^10^, ^11^, ^12^, ^13^ Key elements that affect diagnosis and response to treatment are mutations in various pivotal genes. In the context of cancer, mutations in key genes can dictate the response to anticancer drugs and chemotherapies which ultimately determines the chances of a given patient to recover. A useful and well‐applied strategy for investigating the gene‐to‐phenotype (i.e., drug resistance) relationship, is via whole‐genome screen. Impressive and elegantly described models of genetic screens in cancer are available.^14^, ^15^, ^16^, ^17^ Indeed, sophisticated genetic engineering methods such as CRISPR/Cas9 allow the parallel knockout of any given gene with relative ease hence providing a way to tie gene to phenotype in a genomic fashion.^18^, ^19^ In a mutated CRISPR/Cas9 library, each cell in the population is typically infected with a single guide RNA (gRNA) targeting a specific gene, ensuring that different cells carry unique mutations, which allows for high‐throughput screening of gene functions across the genome. An extensive effort has been invested in gene‐drug response using cancer cell lines (CCLs).^20^ While informative, CCLs' inevitable acquisition of uncontrolled and uncharacterised mutations, is complicating both the interpretation and generalisation of the results of the genetic screening.^21^ An alternative platform for cancer‐relevant resistance gene identification is the application of normal and genetically stable human pluripotent stem cells (hPSCs). Pluripotent cells share a substantial similarity in cellular and molecular features with cancer cells and therefore can be used as a uniform and controllable cancer research platform.^21^, ^22^, ^23^, ^24^ A further efficient development of such a platform is the use of haploid stem cells. Mammalian haploid cells allow the investigations of even recessive alleles in a ‘yeast genetics’ fashion.^25^ Utilisation of such platforms in mice,^26^, ^27^ rats,^28^ monkeys,^29^ and humans^30^, ^31^ haploid pluripotent cells provided valuable insights for various traits and cellular processes. This recent development of the human haploid embryonic stem cells (ESCs) screening platform has opened exciting avenues for basic and applied research alike.^25^, ^32^, ^33^, ^34^, ^35^, ^36^, ^37^, ^38^ To gain a comprehensive view of genes that affect or regulate paclitaxel and carboplatin resistance, we performed genome‐wide loss‐of‐function (LoF) screens. Other genome‐wide mutagenesis systems such as the piggyBac method have yielded interesting findings in mammalian haploid ESCs.^26^, ^39^, ^40^, ^41^, ^42^, ^43^, ^44^. Nevertheless, the selection of the CRISPR/Cas9 method relied on its substantial advantages: high knockout (KO) efficiency, targeting precision, minimal off‐target effects, versatility, easy multiplexing, comprehensive genome coverage, easy target identification (gRNA serves as barcode) and high experimental reproducibility. Collectively, these advantages are aimed at providing a superb tool for LoF screens.^45^ Both paclitaxel and carboplatin screens have yielded selected plausible gene lists and pathways relevant to resistance prediction. Application of the findings from our screens on patients' tumours, enabled the separation of sensitive vs. resistant patients thus, providing a prediction tool. As the anticancer drugs arsenal can offer alternatives in case a certain drug exhibits resistance, an early prediction can provide a significant advantage and may improve treatment. Our results assist this unmet need for the ability to predict whether a patient will respond to the treatment and may have an immediate clinically actionable application.

## 2 RESULTS

### 2.1 Genome‐wide CRISPR LoF screen for paclitaxel and carboplatin resistance

**FIGURE 1:**
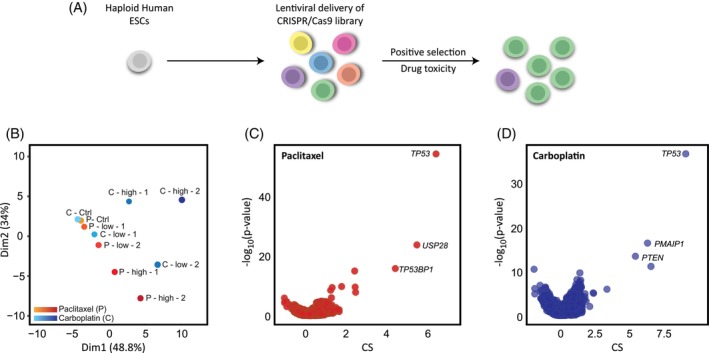
Analysis of whole genome screens identifying resistance to paclitaxel and carboplatin. (A) Schematic illustration of the CRISPR/Cas9 induced mutation library screens to identify genes conferring resistance to chemotherapies. (B) PCA plot demonstrating the collected samples for the paclitaxel and carboplatin screens. Designated orange‐to‐red (paclitaxel) and azure‐to‐blue (carboplatin) shades represent each drug's samples and concentrations. (C) Volcano plot displaying the paclitaxel CRISPR score (CS = log2 fold change) and P‐values for all the genes in the screen. (D) Volcano plot displaying the carboplatin CRISPR score (CS = log2 fold change) and *p*‐values for all the genes in the screen.

Paclitaxel and carboplatin are the most frequently used drugs as first‐line chemotherapy regimens. Despite the effectiveness of these drugs for many oncological patients, they have limited efficiency due to drug resistance, which presents a major unmet clinical impediment.^46^ To uncover genes and mutations that play a role in the resistance of tumours to paclitaxel and carboplatin, we have exploited our haploid hESC LoF genome‐wide library^32^, ^35^ (Figure 1A). We initially calibrated a dose–response curve for the two drugs to select optimal concentrations (moderate and high) for the screens (Figure S1A,B). Then, the haploid hESC—CRISPR/Cas9 LoF library was exposed to the selected concentrations of the two drugs to allow capturing a comprehensive view of the drug‐resistant genetic profile (Figure S1C,D). Cells were monitored daily and upon recovery, they were harvested for DNA extraction and replated (3:1 ratio). Once the replated cells recovered, they were re‐exposed to the drug for additional selection. By the end of the experiment, we obtained 4–6 library samples for sequencing alongside their respective controls (Figure S1C,D). Our library's gRNA abundance between the control and treatments was analysed via our CRISPR Score (CS) analysis pipeline.^32^, ^35^, ^37^ As described previously,^32^ we have taken advantage of an optimised human CRISPR/Cas9 mutated library targeting the genes in the haploid stem cells. This library has been preserved in order to capture a comprehensive repertoire of the targeted genome. Indeed, following the analysis of the control‐untreated library (i.e., ‘time zero’) exhibited a representation of all 18,166 targeted genes for both paclitaxel and carboplatin. For the paclitaxel and carboplatin libraries, an average of 8.76 and 8.65 gRNA per gene were represented, respectively. Principal component analysis (PCA) was performed, showing clear and significant divergence of the treated samples from the controls for both paclitaxel and carboplatin treatments (Figure 1B). This effect was more pronounced for higher concentrations (paclitaxel) or later time points (carboplatin), which may indicate a different selective pressure (Figure 1B). The selected candidate genes were derived from our haploid screens using our CRISPR Score values to highlight the top genes as candidates for conferring resistance. We have focused on enriched genes that have met our statistical threshold (CS > 0.5, *p* < 0.05), suggesting a possible drug‐resistant effect of the gene's null mutation (Figure 1C,D). For further analyses, 50 significantly enriched genes from the paclitaxel screen and 11 genes from the carboplatin screen were selected (see Tables S1 and S2).

**FIGURE 2:**
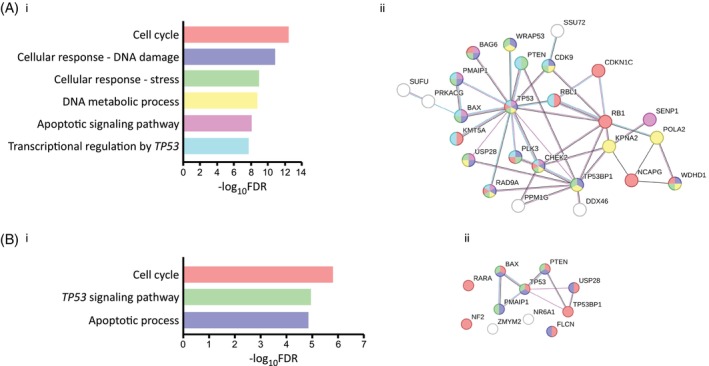
Functional analysis of hESCs whole genome screens. (A) Pathway enrichment of candidate genes. The top 50 genes from the paclitaxel screen were analysed for enrichment and protein interactions using the GSEA and STRING resources, respectively. Colours in STRING represent identified pathways. (B) Pathway enrichment of candidate genes. The top 11 genes from the carboplatin screen were analysed for enrichment and protein interactions using the GSEA and STRING resources, respectively. Colours in STRING represent identified pathways.

We have tested the selected genes for enriched pathways and protein interactions using Gene Set Enrichment Analysis (GSEA) (https://www.gsea-msigdb.org/gsea/index.jsp) and STRING (https://string-db.org/). The 50 top candidate genes from the paclitaxel screen have presented prominent enrichment for cell cycle, apoptosis, DNA damage, stress and the *TP53* transcriptional regulation pathways (Figure 2A‐i). Those pathways appear in a tight protein network, highlighting p53 at its centre (Figure 2A‐ii). Nevertheless, p53 appears to share different pathways with different proteins indicating the versatile role of p53 in different pathways and cellular mechanisms, each capable of providing resistance to paclitaxel (Figure 2A‐ii). Likewise, the shorter list of genes from the carboplatin screen provided concrete indication for the main role of both *TP53* and phosphatase and tensin homologue (*PTEN*) as key candidates for carboplatin resistance (Figure 2B‐i,ii).

Our screens yielded a substantial number of plausible candidate genes. Due to their pivotal role and high abundance in tumours, *TP53* and *PTEN* are the most prominent candidates for individual validation, that is, the test gene's LoF versus wild type (WT) allele.

### 2.2 *TP53* LoF effect on paclitaxel and carboplatin resistance

**FIGURE 3:**
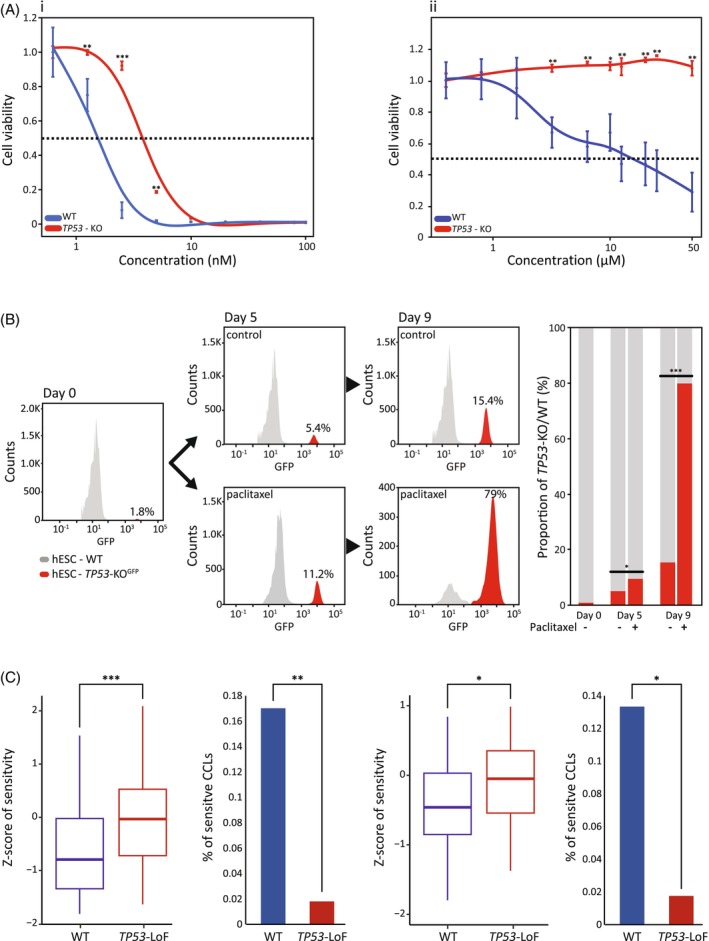
Individual validation of selected candidate genes. (A) Response of *TP53*‐KO hESCs to paclitaxel or carboplatin treatments compared to WT cells. *TP53*‐KO cells exhibit significantly reduced sensitivity to paclitaxel treatment (i) and drug resistance to carboplatin (ii). X‐axis = Concentration (nM and μM, respectively), *Y*‐axis = Cell viability. (B) WT/*TP53*‐KO cell competition assay. A mixture of WT and *TP53*‐KO hESCs was used to test the lack of *TP53* effect on paclitaxel resistance. Plates were seeded with 50:1 ratio of WT/*TP53*‐KO cells in duplicates, then treated with paclitaxel. Changes in *TP53*‐KO‐GFP hESCs were measured by FACS. Summary of all FACS results represented in grey (WT)/red (*TP53*‐KO) bars. (C) Bioinformatic response analysis of (CCLs) to paclitaxel and carboplatin—box plot of *TP53*‐LoF CCLs (*n* = 55) are marked in red, and the WT CCLs (*n* = 47) are marked in blue. The lower the Z‐score is, the more sensitive to the drug the CCLs are. The bar plot is the percentage of sensitive CCLs (Z‐score < −1.5) in the *TP53*‐LoF CCLs (red bar) and in the WT CCLs (blue bar). The left box plot and bar plot show paclitaxel results, and the right box plot and bar plot show the carboplatin results. *p < 0.05; **p < 0.005; ***p < 0.0001.

The information on *TP53’*s essential role in various cell processes, such as growth, survival, and maintenance, is well documented.^47^ *TP53* LoF may confer resistance via growth advantage or reduced sensitivity, however, it doesn't confer resistance to all chemotherapies.^35^ We thus tested whether *TP53* LoF may reduce cells' sensitivity to paclitaxel and carboplatin. We first analysed the sensitivity to paclitaxel and carboplatin by comparing the response of haploid hESC versus haploid *TP53*‐KO cell lines. The toxicity experiment exhibits a clear and significant effect of the *TP53*‐KO on cell survival (Figure 3A). *TP53*‐KO hESCs showed decreased sensitivity to paclitaxel with a significant effect compared to WT cells (IC50 = 3.4 nM and 8 nM, respectively) (Figure 3A). Interestingly, *TP53*‐KO presents dramatic resistance to carboplatin when comparing WT cells to *TP53*‐KO cells (IC50 = 15 uM and >50 uM, respectively) (Figure 3A). In addition, we used our WT/*TP53*‐KO cell competition assay^35^ to test the *TP53* culture‐growth‐advantage effect versus drug sensitivity. Briefly, we mixed the WT hESCs with *TP53‐*KO‐GFP hESCs (98:2 ratio) and tested cell percentage dynamics in paclitaxel untreated and treated plates. Competition dynamics were monitored by fluorescence‐activated cell sorting (FACS) (Figure 3B). The percentage of untreated *TP53*‐KO cells increased from 2% to 15% on day 9, demonstrating that, as expected, *TP53*‐LoF mutation confers a growth advantage. Moreover, the percentage of *TP53*‐KO cells increased dramatically to nearly 80% of the paclitaxel‐treated cells (Figure 3B). This clearly shows that *TP53*‐LoF mutation induces both growth advantage and reduces sensitivity to paclitaxel.

### 2.3 *PTEN* LoF effects on paclitaxel and carboplatin resistance

*PTEN* gene is well known as a tumour suppressor that is frequently mutated in a large array of cancers.^48^ *PTEN* was enriched in both the paclitaxel and carboplatin screens (CS = 1.0, 5.2, FDR = 3.3e−18, 5.8e−28, respectively, Figure 1C,D), however the vast majority of tumour suppressor genes didn't confer resistance to paclitaxel and carboplatin in our system. We therefore tested WT and *PTEN*‐mutated cells for their sensitivity upon treatment with either carboplatin or paclitaxel. *PTEN*‐mutated hESCs showed a significant decrease in sensitivity to carboplatin compared to WT (IC50 = 1.2 μM and 4.8 μM, respectively). When treated with paclitaxel, the *PTEN* mutant exhibited a mild decrease in sensitivity compared to WT (IC50 = 0.8 nM and 1.18 nM, respectively, Figure S2). Taken together, a null mutation in the *PTEN* gene reduced the haploid hESC sensitivity to both carboplatin and paclitaxel, which correlates well with the effect found in our whole genome screens.

### 2.4 Validation in tumour models of genes involved in drug‐resistance

As our platform is based on hESCs, we next tested the effectiveness of paclitaxel and carboplatin on the *TP53* mutant cancer model. Thus, we tested the sensitivity of various CCLs to the drugs. We used data from Cancer Target Discovery and Development (CTD2) (https://www.cancer.gov/ccg/research/functional-genomics/ctd2) which contains sensitivity measures of CCLs to different drugs. We compared the sensitivity of paclitaxel and carboplatin to *TP53*‐LoF CCLs (*n* = 55) and CCLs without mutations in *TP53* (*n* = 47). As shown in Figure 3C, there is a significant difference in the sensitivity of *TP53*‐LoF CCLs and other CCLs for both paclitaxel and carboplatin (T‐test; *p* = 0.00047 and *p* = 0.0081, respectively). Next, we examined the differences in the proportion of sensitive CCLs between *TP53*‐LoF and lines without mutations in *TP53*. We defined sensitive CCLs as those having a Z‐score lower than −1.5 (*n* = 9). We found that for both paclitaxel and carboplatin, there is a higher proportion of sensitive CCLs without mutations in *TP53* compared to those with *TP53*‐LoF (proportion *Z*‐test; *p* = 0.009 and *p* = 0.029, respectively).

To perform retrospective validation on human tumours, we used The Cancer Genome Atlas (TCGA) database (https://www.cancer.gov/ccg/research/genome-sequencing/tcga). We tested whether a mutation profile can predict a patient's response to treatment with either paclitaxel or carboplatin, based on the genetic predictions arising from the genome‐wide screening in hESCs. We thus crossed the mutation data and clinical information, with the treatment type and the patients' response. About 222 patients treated with paclitaxel were divided according to their response to treatment: patients with complete response or partial response were classified as sensitive to treatment with paclitaxel (*n* = 156), whereas patients with stable disease or clinical progressive disease were classified as resistant to paclitaxel (*n* = 66). Likewise, of the 243 patients who were treated with carboplatin, 158 were sensitive to the treatment and 85 were resistant.

**FIGURE 4:**
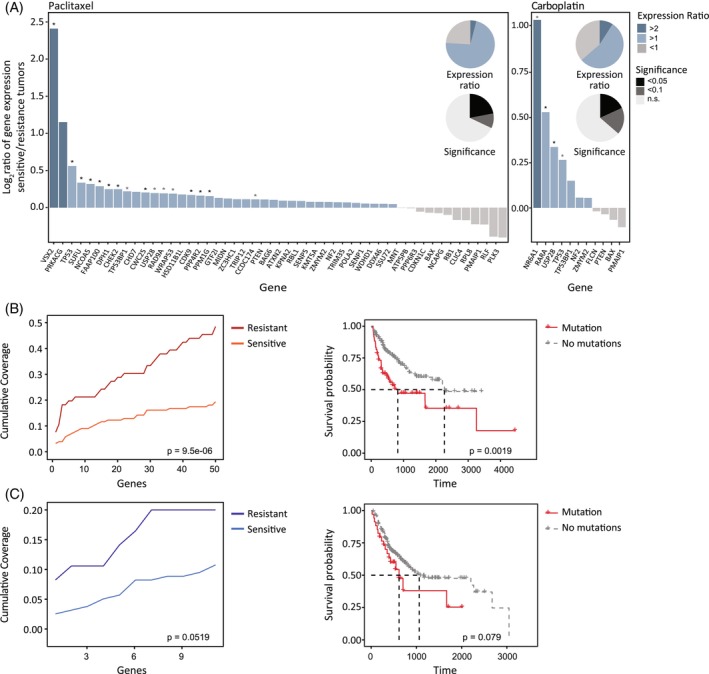
Analysis of genes involved in drug resistance in human tumours. (A) Left: Paclitaxel‐resistant mutated genes, ordered by fold change of expression (TPM) between resistant and sensitive paclitaxel‐treated TCGA samples. Bar colours indicate log2 fold change per gene, showing lower expression among resistant patients in 39/50 genes. Asterisks mark statistical significance of the mean difference between the group, FDR < 0.05 for 11 out of 50 genes, and FDR < 0.1 for another 18 out of 50 genes. Pie charts colour‐matched to illustrate ratio of top gene list. Right, same data shown for carboplatin‐resistant mutated genes. 7/11 showing lower expression in resistant mutants, with FDR < 0.05 for 2 out of 11 genes, and FDR < 0.1 for another 4 out of 11 genes. (B) Cumulative coverage plot of top 50 genes in paclitaxel (left) and Kaplan Meier plots of patients with or without mutations in at least one of the paclitaxel genes (right). (C) Cumulative coverage plot of top 11 genes in carboplatin (left) and Kaplan Meier plots of patients with or without mutations in at least one of the carboplatin genes (right).

Initially, we examined whether the genes that their KO enabled hESCs to be resistant to either paclitaxel or carboplatin were downregulated in tumours that showed resistance to the drugs. We have thus compared the expression of each of the 50 genes that are involved in paclitaxel resistance in tumours that are either sensitive or resistant to the drug. Our data showed that 76% (38/50) of the potential genes are indeed downregulated in the resistant tumours (Figure 4A‐left). For about 40% (15/38) of the downregulated genes, the effect was significant, with *p* < 0.1 (Figure 4A‐left). Similar results were obtained for carboplatin; our data showed that 64% (7/11) of the potential genes were indeed downregulated in the resistant tumours (Figure 4A‐right). For 71% (5/7) of the downregulated genes, the effect was significant, with *p* < 0.1 (Figure 4A‐right).

Next, we tested the mutational profile of the paclitaxel‐resistant genes. The genes were divided into two groups based on their CRISPR Score values, that is, Group 1: 0.5 < CS < 5 and Group 2: CS ≥ 5. These thresholds were defined by analysing the distribution of the CRISPR Scores. The CRISPR Scores of the 50 genes range from 1.4 to 7.15, with a clear cut‐off between the top 3 (*TP53*, *TP53BP1*, and *USP28*) and the other 47 genes. Therefore, we created a group defined by the max CS of a patient and can be divided into 3 groups: patients without mutations in any of the 50 genes, patients with mutations in genes with 0.5 < CS < 5, and patients with mutations in genes with CS > 5. As shown in Figure S2D, out of 66 patients that were resistant to paclitaxel the algorithm has identified 12 and 20 patients with high and moderate likelihood to be resistant to paclitaxel, respectively. Out of 156 patients that were sensitive to paclitaxel the algorithm has identified 6 and 24 patients with high and moderate likelihood to be resistant to paclitaxel, respectively. In 126 of the sensitive patients, no mutations that confer resistance to paclitaxel, according to the algorithm, were found.

The proportion of resistant subjects identified by the algorithm in the resistant group is 48% (32/66) and 19% (30/156) in the sensitive group (Figure 4B‐i). A Pearson's chi‐squared test on the proportions of the percentage of identified resistant vs. sensitive, reached statistical significance with a *p* < 0.0001. The odds ratio obtained between the proportions was 3.95 with a 95% confidence interval of (2.11, 7.39). An odds ratio of 3.95 means the odds of having a mutation in one of the 50 genes from the list employed by our algorithm is 3.95 times higher among the resistant group than the sensitive group.

We applied a logistic regression model to test the ability of the predefined mutations to predict the outcome of paclitaxel treatment. The predictor variable in our case is the Max_CS and patients harbouring mutations in genes with CS > 5 are predicted to be more likely to be resistant to paclitaxel. According to the logistic regression model, the probability of being resistant for a patient without a mutation is 0.21. The probability of being resistant for a patient with a mutation (Group 1) is 0.44. The probability of being resistant for a patient with a mutation (Group 2) is 0.69. The logistic regression model reached a significance level of 9.50e−06.

The same analysis was performed on carboplatin with data from 243 patients, where 158 were sensitive to the treatment and 85 were resistant. We tested the mutational profile of 11 genes, with the same division to groups according to the CRISPR Score as described for paclitaxel. As shown in Figure S2, out of 85 patients that were resistant to Carboplatin the algorithm has identified 9 and 8 patients with high and moderate likelihood to be resistant to carboplatin, respectively. Out of 158 patients that were sensitive to carboplatin the algorithm has identified 8 and 9 patients with high and moderate likelihood to be resistant to carboplatin, respectively. In 141 of the sensitive patients, no mutations that confer resistance to carboplatin, according to the algorithm, were found. The proportion of resistant subjects identified by the algorithm in the resistant group is 20% (17/85) and 11% (17/158) in the sensitive group (Figure 4B‐ii). According to the logistic regression model we applied, the probability of being resistant for a patient without a mutation is 0.33. The probability of being resistant for a patient with a mutation (Group 1) is 0.43. The probability of being resistant for a patient with a mutation (Group 2) is 0.55. The logistic regression model reached a significance level of 0.0519.

To further validate the ability of the mutational profile based on the 50 genes to confer resistance to paclitaxel, we performed a survival analysis. The 222 patients were divided into groups according to whether they had a mutation in at least one of the 50 genes. Figure 4B shows the Kaplan–Meier plot of these groups. As can be seen in the figure, patients with a mutation in at least one of the 50 genes have a significantly lower survival rate compared to patients without a mutation in any of the genes (log‐rank test; *p* = 0.0019). The same analysis was performed for patients treated with carboplatin, where patients were divided according to their mutational profile in the 11 genes. We found that patients with a mutation in at least one of the 11 genes have a lower survival rate compared to patients without any mutation in these genes (Figure 4C; Log‐rank test; *p* = 0.079).

## 3 DISCUSSION

This project aims at defining core genes that enable the prediction of resistance to two widely used anticancer drugs—paclitaxel and carboplatin. We used our unique haploid hESC—CRISPR/Cas9 library as an alternative robust approach for the identification of genes that confer resistance. Previously, we have demonstrated that the molecular processes shared by both hESCs and cancer cells reinforce the application of hESCs in genome‐wide screening for drug‐resistant mutations.^35^ ESCs share key similarities with cancer cells, including their capacity for unlimited self‐renewal driven by certain shared molecular pathways. However, despite these similarities, ESCs may not fully capture the heterogeneity and genetic instability of tumour cells. The use of haploid hESCs has an advantage in LoF screening over CCLs, as haploid cells have only one copy of each gene versus aneuploid CCLs, which may have multiple copies, resulting in trisomy, tetrasomy and even up to octosomy.^49^, ^50^ To achieve efficient LoF via nonsense mutations, a 1–2 base pair frameshift is typically required, preferably in the early exons of the target gene. The gRNAs used in this study's CRISPR library were specifically designed to target these regions. In haploid ESCs, there is a high probability of inducing a frameshift in the single allele of each targeted gene. This probability decreases as the number of alleles increases, which is particularly relevant when working with CCLs as they are often aneuploid, leading to an increased number of alleles. Haploid hESCs do not carry any significant mutation,^30^, ^51^ while human CCLs harbour mutations in multiple genes, which makes the interpretation of the results very challenging. A similar path was taken recently by using stable diploid pluripotent cells and creating a ‘local haploid’ region^21^ further highlighting the value of haploid cells in cancer research. It is important to note that the mutation landscape of tumours is highly complex and rapidly changes as more and more mutations are accumulated. Therefore, a complete capture of all relevant mutations represents a serious challenge. However, a pivotal class of mutations that occur in various cancers and confer resistance are LoF mutations that inactivate key tumour suppressor genes. The mutagenesis strategy used by us created high LoF mutations in the haploid stem cells library, in accordance with LoF mutations in tumours. Our system efficacy was first exemplified by the clear PCA analysis of control and treated samples with distinctive differences based both on the given concentration and duration (Figures 1B and S1). Those results allow us to further refine and prioritise genes that present significant enrichment (Figure 1C,D). *TP53* appears as the most significant gene which well correlates to previous studies tying *TP53* mutations and drug resistance.^35^, ^52^, ^53^, ^54^, ^55^, ^56^ However, we aimed to use *TP53* as an anchor gene alongside other genes for the purpose of predicting possible resistance, enabling a calculated treatment regimen. The paclitaxel screen provided a core list of 50 genes that we took for further in‐depth analysis. Analysing pathways enrichment by GSEA of the genes provided five major pathways (Figure 2A‐i). We have used the STRING dataset to visualise interactions between the different genes. Interestingly, a large proportion of the genes interact or share common pathways suggesting that beyond the resistance genes per se, we can view drug resistance at a pathway level (Figure 2A‐ii). Parallel analysis of the carboplatin screen provided a shorter list of plausible genes (11 genes, Figure 2B‐ii). Enrichment analysis indicated three pathways for which *TP53*, *PTEN*, and *BAX* are central (Figure 2B‐i,ii). It is worth noting that while the *TP53* gene indeed has a central role in drug resistance, the highlighted pathways suggest that different combinations of genes, for which no prior connection to paclitaxel or carboplatin was known, confer resistance as well.

Individual validations for *TP53* and *PTEN* (key player in cancer and the p53 pathway) revealed additional data for their role in resistance. Carboplatin induces DNA damage, while paclitaxel disrupts cell division by stabilising microtubules. Resistance to carboplatin often involves improved DNA repair whereas paclitaxel resistance usually stems from changes in apoptotic pathways. These differences in action may lead to distinct resistance mechanisms for each drug. Optimising drug concentrations relied on careful monitoring of the effect on our cells and selecting the appropriate concentration that exhibits massive cell death and yet allows cell recovery (Figure S1). The response differences shown in Figure 3A (very significant resistance of p53‐KO to carboplatin and less to paclitaxel) may represent the different resistance mechanisms to the drugs that arise from the p53 KO mutation. When tested individually, haploid hESCs with *TP53*‐KO indeed present an impressive decrease in sensitivity to paclitaxel (Figure 3A‐i). Using a cell competition assay that has been successfully tested,^35^ we could separate the effect of *TP53*‐KO cells to prevail in culture, from its additional effect following paclitaxel treatment in a course of nine days (Figure 3B). This phenomenon suggests independent mechanisms by which *TP53*‐KO cells (or *TP53* LoF cells) are coping with changes in their environment. When tested for carboplatin resistance, *TP53*‐KO hESCs present a dramatic staggering resistance showing no response to the treatment (Figure 3A‐ii). When *PTEN*‐KO hESC was treated with paclitaxel, we found a marginal difference in sensitivity and a discrete number of genes (where *TP53* and *PTEN* are the significant players) while the resistance to paclitaxel may require many more partners besides *TP53* and *PTEN* (Figure 2). In the future, additional genes and pathways can be further validated for their role in the resistance to paclitaxel and carboplatin. An independent bioinformatic analysis on CCLs provided further support for our findings. Separating CCLs by their *TP53* mutation status (i.e., WT vs. LoF), we found a significant difference in response to paclitaxel and carboplatin between the groups with *TP53*‐LoF presenting less sensitivity and their fraction among all sensitive CCLs approaching zero (Figure 3C). The results from our in vitro screens, validations, and CCLs analysis, encouraged us to test them against existing patient tumour information, thus using their potential to provide valuable predictions with clinical benefits.

To evaluate the prognostic power of our findings, we have performed a retrospective analysis on patient data derived from the TCGA as it contains a substantial number of patients' tumour profiles. Testing our paclitaxel genes list against patients' specific expression patterns, revealed that even a core list of 15 genes can predict sensitivity or resistance to the treatment. Patient tumours presenting low or no expression of the genes (i.e., LoF) were resistant to paclitaxel treatment (Figure 4A). The carboplatin genes list, though containing fewer genes, provided a putative core of five genes that enable to distinguish between resistant and sensitive patients based on expression. Parallel to the expression‐based prediction analysis, an independent complementary predictive analysis algorithm based on gene mutations allowed us to significantly predict a patient being resistant to either paclitaxel or carboplatin (Figure 4B). The robust predictive power achieved via genes/pathway analysis, emphasises the advantage of gene combinations as a useful tool for future precision treatment. Survival analysis further confirmed that indeed patients with mutations in the genes provided by the screen, present low survival rates (Figure 4B).

The results presented in this study, demonstrate the importance of a genome‐wide identification of anticancer resistance genes. Harnessing the advantageous feature of haploid hESCs, we were able to identify tightly interacting genes that, in turn, are valuable for patient classification as resistant or sensitive. Mutation data can be used to subject a patient to the best possible treatment available and refrain, as much as the medical treatment regimen allows, from using anticancer drugs that will have minimal or no effect. Finally, our presented work suggests clinical implications for patient treatment considerations. Predicting which patient is probable to be resistant to either of the tested drugs can lead to a more efficient anticancer drug selection and higher efficacy in treatment.

## 4 MATERIALS AND METHODS

### 4.1 Cell lines and culture

The following cell lines were used in this study: Haploid hESCs^30^ and h‐pES10‐based mutant library recently established by us.^32^ Diploid hESC‐TP53 LoF mutation with GFP‐tagged tubulin—TUB::GFP.; female 293T cells, obtained from R. Weinberg (Whitehead Institute). The library of mutated hESC was cultured at 37°C and 5% CO_2_ on matrigel‐coated plates (Corning) in feeder‐free mTeSR1 (STEMCELL Technologies) medium supplemented with 10 μM ROCK inhibitor Y‐27632 (Stemgent) for one day after splitting. Before reaching confluency, cells were passaged by quick trypsinisation using TrypLE Select (Thermo Fisher Scientific) and plated in feeder‐free conditions. WA09 hESCs were cultured on feeder layer growth‐arrested mouse embryonic fibroblasts (MEFs) in standard hESC growth medium, composed of KO Dulbecco's modified Eagle's medium (DMEM) supplemented with 15% KO serum replacement (KSR, Thermo Fisher Scientific), 2 mM L‐glutamine, 0.1 mM nonessential amino acids, 50 units/mL penicillin, 50 mg/mL streptomycin, 0.1 mM β‐mercaptoethanol and 8 ng/mL basic fibroblast growth factor (bFGF) for maintenance and during the generation of KO cell lines. MEFs and 293T cells were cultured in DMEM supplemented with 10% foetal bovine serum (Invitrogen), 2 mM L‐glutamine, 50 units/mL penicillin and 50 mg/mL streptomycin. All hESC lines in this study, were used under the Israeli guidelines concerning hESC research (http://bioethics.webcare.org.il/english/report1/Report1-e.html).

### 4.2 Drug calibration

Ten different drug concentrations were tested for each drug in triplicates. hESC cells were grown on 96 well plates. hESC density was 15,000 cells per well. Six plates were used to allow six time points per concentration per drug. The drug was added on day ‘0’ and the medium was replaced every 24 h. Cell viability was assessed by a CellTiter‐Glo luminescent cell viability assay according to the manufacturer's instructions (Promega). Cell viability was monitored at the following time points‐ day 1, 2, 3, 6, 10, and 13. Luminescence intensity was normalised to control conditions, and triplicates were averaged. This comprehensive calibration regime allowed a careful selection of concentrations that produce significant cell death yet allow cell recovery. The drugs in this study; paclitaxel and carboplatin, were purchased from Cayman Chemical (Ann Arbor, Michigan, USA). Preparations of all drugs were done according to vendor protocols.

### 4.3 CRISPR screen of anticancer drugs resistant genes

Haploid hESC—CRISPR/Cas9 LoF library cells were thawed on 10 cm Matrigel‐coated plates. Upon confluency, cells were harvested, counted and re‐seeded for each experiment. On the following day, control plates were harvested for DNA extraction and served as control. For the remaining plates, we replaced the medium with fresh mTeSR containing the drugs in the preselected concentrations. Cell death and recovery were carefully monitored throughout the different experiments. Once cells recovered from the anticancer drug treatment, they were harvested for DNA extraction and re‐seeded for an additional round of drug treatment and DNA extraction.

### 4.4 DNA extraction, PCR amplification of sgRNAs and high‐throughput DNA sequencing

Genomic DNA was extracted with a Blood & Cell Culture DNA Midi Kit (QIAGEN) or a gSYNC DNA extraction kit (Geneaid) according to the manufacturer's instructions. The locus containing the sgRNA integration was amplified with the following primers, which also contain overhang sequences compatible for Nextera DNA library preparations (Illumina):

$$5^{\prime }-\text{TCGTCGGCAGCGTCAGATGTGTATAAGAGACAGG}\text{GCTTTATATATCTTGTGGAAAGGACG}-3^{\prime }\;\left(\text{forward}\right)$$

$$5^{\prime }\text{GTCTCGTGGGCTCGGAGATGTGTATAAGAGACAG}\text{ACGGACTAGCCTTATTTTAACTTGC}-3^{\prime }\;\left(\text{reverse}\right),$$

Or

$$5^{\prime }\text{TCGTCGGCAGCGTCAGATGTGTATAAGAGACAGNN}\text{NNNNNNNNGGCTTTATATATCTTGTGGAAAGGACG}\;3^{\prime }\;\left(\text{forward}\right)$$

$$5^{\prime }\text{GTCTCGTGGGCTCGGAGATGTGTATAAGAGACA}\text{GACGGACTAGCCTTATTTTAACTTGC}\;3^{\prime }\;\left(\text{reverse}\right).$$

The total genomic DNA for each time point was divided into 50 μL PCR reactions with 4 μg DNA input. The PCR settings were as previously described.^32^ After purification of the 160‐base‐pair (bp) products, a second PCR reaction was performed using Nextera adapter primers to generate a Nextera DNA library according to the manufacturer's instructions (Illumina). DNA libraries containing sgRNA constructs from two replicate experiments were sequenced using NextSeq 500 (Illumina).

### 4.5 Analysis of CRISPR/Cas9 library results

DNA next‐generation sequences were aligned to the sgRNA sequences with the bowtie2 program,^57^ allowing up to one mismatch. For the volcano plots presented in Figure 1C,D, the count table was normalised to the total number of reads in each of the specific drug concentrations, and replicates were averaged. CRISPR scores (CS) are the average of the log_2_ ratios of the abundance of all sgRNAs for each gene between treated and control samples. Statistical significance was determined by the Kolmogorov–Smirnov test using ks_2samp from Python's SciPy. Stats module as previously described.^32^ In doing so, the ratio of sgRNAs for every gene was compared between treated and control samples. The Benjamini–Hochberg FDR correction was accomplished with the multiple test features from Python's statsmodels.sandbox.stats.multicomp module. For the analysis described in the paper and shown in Tables S1 and S2, the count table was normalised, and CS, *p*‐value and FDR were calculated using Model‐based Analysis of Genome‐wide CRISPR/Cas9 KO (MAGeCK).^28^, ^44^ We applied inclusion/exclusion criteria of genes based on the number of sgRNAs—first, genes with a count of non‐zero guides in the control less than four were excluded from the analysis. Then, genes were included if they: (1) had more than 3 non‐zero guides in the treatment, (2) had 3 non‐zero guides in the treatment and a count of non‐zero guides in the control ranging from 4 to 7, or (3) having 2 non‐zero guides in the treatment and a count of non‐zero guides in the control ranging from 4 to 5. In the final step, a threshold of CS > 0.5 and *p* < 0.05 was applied.

### 4.6 CCL analysis

Sensitivity data of CCLs treated with paclitaxel or carboplatin was downloaded from CTD2 (https://www.cancer.gov/ccg/research/functional-genomics/ctd2). To categorise CCLs as *TP53*‐LoF or WT we applied specific inclusion criteria—for *TP53*‐LoF: (1) Presence of an LoF mutation, defined as a frame‐shift deletion, frame‐shift insertion, or nonsense mutation, (2) Percentage alteration exceeding 80%, and (3) IC50 value for Nutlin treatment exceeding 50. Conversely, to classify a CCL as WT, the following inclusion criteria were applied: (1) Absence of a *TP53* mutation, and (2) IC50 value for Nutlin treatment below 10.

### 4.7 Retrospective data analysis

Retrospective validation on human tumours, performed using data from the TCGA database. RNA‐seq data of all the patients treated with paclitaxel or carboplatin was downloaded and TPM values used for the analysis. In addition, we gathered clinical information available for all patients from the TCGA, including the different treatments they received and the response to these treatments, as well as their mutational profile as detailed in the mutation annotation format (MAF) files. We applied several inclusion criteria based on the clinical data: (1) patients with less than 1,000 mutations, (2) Patients who were treated with a protocol that includes paclitaxel or carboplatin, (3) Patients with available genetic information and with known response to treatment with paclitaxel or carboplatin. Next, we applied inclusion criteria on the mutations data: (1) Mutations that are defined as one of the following: Frame_Shift_Del, Nonsense_Mutation, Frame_Shift_Ins, Splice_Region, In_Frame_Ins, Splice_Site, In_Frame_Del, Translation_Start_Site, Missense_Mutation, (2) Mutations with read depth >20, (3) mutations with t_alt /t_depth > 10%, (4) For highly mutated genes(genes that are mutated in more than 3.5% of all patients), only LoF mutations were kept (Frame_Shift_Del, Nonsense_Mutation, Frame_Shift_Ins) with % alteration >80%, (5) Mutations that occur in nuclear genes.

### 4.8 Generation of KO cell lines

About 1–3 sgRNAs per gene were used to target candidate genes. *TP53* sgRNA was cloned into the PX458 CRISPR/CAS9 vector (Addgene cat. no. 48138). hESC was transfected in plates using the X‐tremeGENE 9 DNA transfection reagent (ROCHE) according to the vendor protocol. Selection for *TP53*‐KO cells was done by adding 10 μM Nutlin‐3 (Caymanchem, 10,004,372–10) in the hESC medium for 10–14 days (about 2 weeks). PTEN sgRNAs were cloned into the lentiCRISPR v2 lentiviral vector (a gift from Feng Zhang, Addgene cat. no. 52961) to produce the lentiviruses, 293 T cells were transfected with sgRNA‐containing lentiCRISPR v2 or sgRNA‐containing lentiCRISPR v2‐Blast, pCMV‐VSV‐G (a gift from Robert Weinberg, Addgene cat. no. 8454) and psPAX2 (a gift from Didier Trono, Addgene cat. no. 12260) plasmids at a ratio of 2:1:1.5 (10 mg total per plate), respectively, in the presence of polyethyleneimine ‘Max’ (PEI‐Max) (Polysciences) at a 1:2 ratio of DNA to PEI‐Max. Transfection medium was exchanged with standard hESC growth medium (described above) after 16–24 h, and lentiviral particle‐containing culture supernatant was harvested 60–65 h after transfection. Culture supernatant was spun down at 3000 rpm for 10 min at 4°C and then filtered through 0.45 mm cellulose acetate filters (Millipore). The filtered supernatant was frozen in aliquots at −70°C. Haploid‐enriched hESC cultures were trypsinised with Trypsin–EDTA, centrifuged and resuspended in hESC growth medium supplemented with 10 μM ROCK inhibitor Y‐27632 and 8 mg ml^−1^ polybrene (Sigma). The thawed viruses were then added to the cell suspension. Transduced cells were plated on feeder layer MEFs. About 24 h after transduction, the virus‐containing medium was replaced with a standard hESC growth medium. About 36–48 h after transduction, the medium of the cells was replaced with a medium that contained puromycin (0.3 mg/mL, Sigma). Cells were kept under antibiotic selection for 7–10 days. sgRNA sequences for the genes *PTEN* and *TP53* are summarised in supplementary Table 3.

In *TP53* mutant cells, a GFP cassette was inserted into the end region of the tubulin gene using CRISPR–HOT method.^58^

Resistance/sensitivity assay for *TP53* was performed in 96 well plates, in triplicates, in a similar manner as the calibration assay. In the *PTEN* validation, drug treatment was readministered every 24 h for the duration of the assay. Paclitaxel and carboplatin were each validated against both *TP53* and *PTEN‐*KO cells.

### 4.9 Cell competition assay

To assess the resistance or insensitivity of *TP53‐*KO to paclitaxel, we performed a cell competition assay. In this assay, GFP‐labelled, *TP53*‐KO cells were mixed in predetermined proportions with WT cells, and changes in the proportions of the labelled cells were measured by FACS analysis. The comparison of control plates (mixed cells with standard medium) versus drug‐treated plates enabled the distinction between a mutation growth advantage and response to drug treatment. TUBB::GFP *TP53*‐KO hESCs and WT hESCs were grown on Matrigel‐coated plates and harvested using TrypLE Select (Thermo Fisher Scientific) upon well confluency. WT and TUBB::GFP *TP53*‐KO cells were mixed in 98%: 2% ratio and were re‐seeded on six‐well plates (day −1). On day 0, cells were harvested and prepared for FACS analysis (below). Control and experiment plates were kept with medium replacement (mTeSR, with or without paclitaxel). The drug concentrations were used in the genome‐wide screen. Cell competition monitoring was performed on Days 0, 5 and 9.

### 4.10 Flow cytometry

Cell samples for GFP expression analysis were spun down at 1500*g* for 4 min and the cell pellet was gently resuspended in 2 mL FACS solution (phosphate‐buffered saline.2% FCS). Live cells were discriminated from cell debris and dead cells based on physical parameters (forward‐ and side‐light scatter). Fluorescence background levels were set with WT cells (GFP‐) cells. Following harvesting, cells were filtered through a 70 μm cell strainer and analysed by flow cytometry (BD Biosciences FACSAria III) and Flowjo software (FlowJo LLC).

### 4.11 Statistical analysis

Statistical analysis was performed using Python, R statistical environment, JMP 15.0.0 software (SAS Institute Inc.) and Microsoft Office Excel. Data are presented as median‐centred. FDR was controlled using the Benjamini–Hochberg correction using an alpha of 0.05 or 0.1 for statistical significance, as indicated. Chi‐square test was applied to test the statistical significance of the difference in the percent of subjects identified by the device as resistant to paclitaxel/carboplatin between the paclitaxel/carboplatin resistant group and the paclitaxel/carboplatin sensitive group. A logistic regression was applied to calculate the Odds Ratio percent (CI 95%) of subjects identified by the device as resistant to paclitaxel/carboplatin between the paclitaxel/carboplatin resistant group and the paclitaxel/carboplatin sensitive group. The objective response rate (ORR) was calculated for all patients and for patients who were identified as resistant by the algorithm.

## AUTHOR CONTRIBUTIONS

Jonathan Nissenbaum, Emanuel Segal, Hagit Philip, Rivki Cashman, Oded Kopper and Nissim Benvenisty designed the experiments; Jonathan Nissenbaum, Emanuel Segal, Hagit Philip and Rivki Cashman, performed the experiments with assistance from Tamar Golan‐Lev, Adi Turjeman and Ofra Yanuka; Jonathan Nissenbaum, Emanuel Segal, Hagit Philip, Rivki Cashman and Oded Kopper wrote the manuscript with input from Benjamin E. Reubinoff, Elyad Lezmi and Nissim Benvenisty; Oded Kopper and Nissim Benvenisty supervised the work.

## FUNDING INFORMATION

This work was partially supported by the Azrieli Foundation, the Israel Science Foundation (2054/22), the ISF‐Israel Precision Medicine Partnership (IPMP) Program (3605/21) and by NewStem Ltd. Nissim Benvenisty is the Herbert Cohn Chair in Cancer Research.

## CONFLICT OF INTEREST STATEMENT

Oded Kopper and Nissim Benvenisty are V.P. R&D and CSO of NewStem Ltd., respectively.

## Supporting information


**Figure S1.** Paclitaxel (A) and carboplatin (B) calibration curves. Haploid hESCs were treated with the drugs for 2 weeks under conditions covering various concentrations. Cell viability was assessed using CellTiter‐Glo assay. Cell death and cell recovery were monitored for optimal concentrations used for the genome‐wide screens (dotted curves). *X* axis = days of treatment, *Y* axis = cell viability. Schematic illustration of the paclitaxel (C) and carboplatin (D) screen treatments. Black bars represent the length of each screen. Coloured arrows are the treatment time point between cell death and cell recovery. Rectangles represent cell passages and library samples for DNA extraction.
**Figure S2**. Response of PTEN‐KO hESCs to paclitaxel or carboplatin treatments compared to WT cells. Cell viability assay of PTEN‐KO and WT hESCs treated with paclitaxel (A) or carboplatin (B). PTEN‐KO cells exhibit moderately reduced sensitivity to paclitaxel treatment. B. PTEN‐KO cells exhibit significant insensitivity to carboplatin treatment. *p < 0.05, **p < 0.001 (C). Left: Cumulative coverage plot of top 50 genes in paclitaxel+carboplatin. Genes are ordered by their CRISPR score. *Y*‐axes are the cumulative coverage of patients with mutations in the genes, in the resistant and sensitive groups. Right: Kaplan Meier plots of patients with or without mutations (yellow or blue lines, respectively) (D). Tables presenting how resistant and sensitive patients are divided across the different groups of patients that we have defined—without mutations (0), patients with mutations in genes with max CS between 1 and 5 (1), patients with mutations in genes with max CS > 5 (2). The tables presented are for paclitaxel (upper table), carboplatin (middle table) and the combination of paclitaxel and carboplatin (lower table).

**Table S1.** 50 significantly enriched genes from the paclitaxel screen.
**Table S2**. 11 significantly enriched genes from the carboplatin screen.
**Table S3**. sgRNA sequences for the generation of knockout cells for PTEN and TP53.

## Data Availability

The data that support the findings of this study are openly available in ArrayExpress at https://www.ebi.ac.uk/biostudies/arrayexpress/studies/E‐MEXP‐2655, reference number E‐MTAB‐13683.
