# Supplementary material for: Predicting tumour resistance to paclitaxel and carboplatin utilising genome‐wide screening in haploid human embryonic stem cells
Source: Cell Prolif. 2024 Nov 10;58(3):e13771. doi: 10.1111/cpr.13771 (PMC11882768; doi:10.1111/cpr.13771)
Supplement: Supplementary file 1 — Figure S1. Paclitaxel (A) and carboplatin (B) calibration curves. Haploid hESCs were treated with the drugs for 2 weeks under conditions covering various concentrations. Cell viability was assessed using CellTiter‐Glo assay. Cell death and cell recovery were monitored for optimal concentrations used for the genome‐wide screens (dotted curves). X axis = days of treatment, Y axis = cell viability. Schematic illustration of the paclitaxel (C) and carboplatin (D) screen treatments. Black bars represent the length of each screen. Coloured arrows are the treatment time point between cell death and cell recovery. Rectangles represent cell passages and library samples for DNA extraction. Figure S2. Response of PTEN‐KO hESCs to paclitaxel or carboplatin treatments compared to WT cells. Cell viability assay of PTEN‐KO and WT hESCs treated with paclitaxel (A) or carboplatin (B). PTEN‐KO cells exhibit moderately reduced sensitivity to paclitaxel treatment. B. PTEN‐KO cells exhibit significant insensitivity to carboplatin treatment. *p < 0.05, **p < 0.001 (C). Left: Cumulative coverage plot of top 50 genes in paclitaxel+carboplatin. Genes are ordered by their CRISPR score. Y‐axes are the cumulative coverage of patients with mutations in the genes, in the resistant and sensitive groups. Right: Kaplan Meier plots of patients with or without mutations (yellow or blue lines, respectively) (D). Tables presenting how resistant and sensitive patients are divided across the different groups of patients that we have defined—without mutations (0), patients with mutations in genes with max CS between 1 and 5 (1), patients with mutations in genes with max CS > 5 (2). The tables presented are for paclitaxel (upper table), carboplatin (middle table) and the combination of paclitaxel and carboplatin (lower table). [file CPR-58-e13771-s001.pdf]

Supplementary information

Predicting tumor resistance to paclitaxel and carboplatin utilizing genome-wide screening in haploid human embryonic stem cells

Jonathan Nissenbaum[1, #, *], Emanuel Segal[2, 3, #], Hagit Philip[1, #], Rivki Cashman[1, #], Tamar Golan-Lev[2], Benjamin E. Reubinoff[3], Adi Turjeman[4], Ofra Yanuka[2], Elyad Lezmi[1], Oded Kopper[1, *] and Nissim Benvenisty[1, 2, *]

[1]NewStem LTD, P.O. Box 39173, Jerusalem 913910, Israel

[2]The Azrieli Center for Stem Cells and Genetic Research, Department of Genetics, Silberman Institute of Life Sciences, The Hebrew University, Jerusalem 91904, Israel.

[3]Hadassah Stem Cell Research Center, Goldyne Savad Institute of Gene Therapy, Department of Obstetrics and Gynecology, Ein Kerem, Hadassah Hebrew University Medical Center, Jerusalem 91120, Israel.

[4]The Center for Genomic Technologies, The Hebrew University, Jerusalem 91904, Israel.

[#]These authors contributed equally

*Co-Correspondence

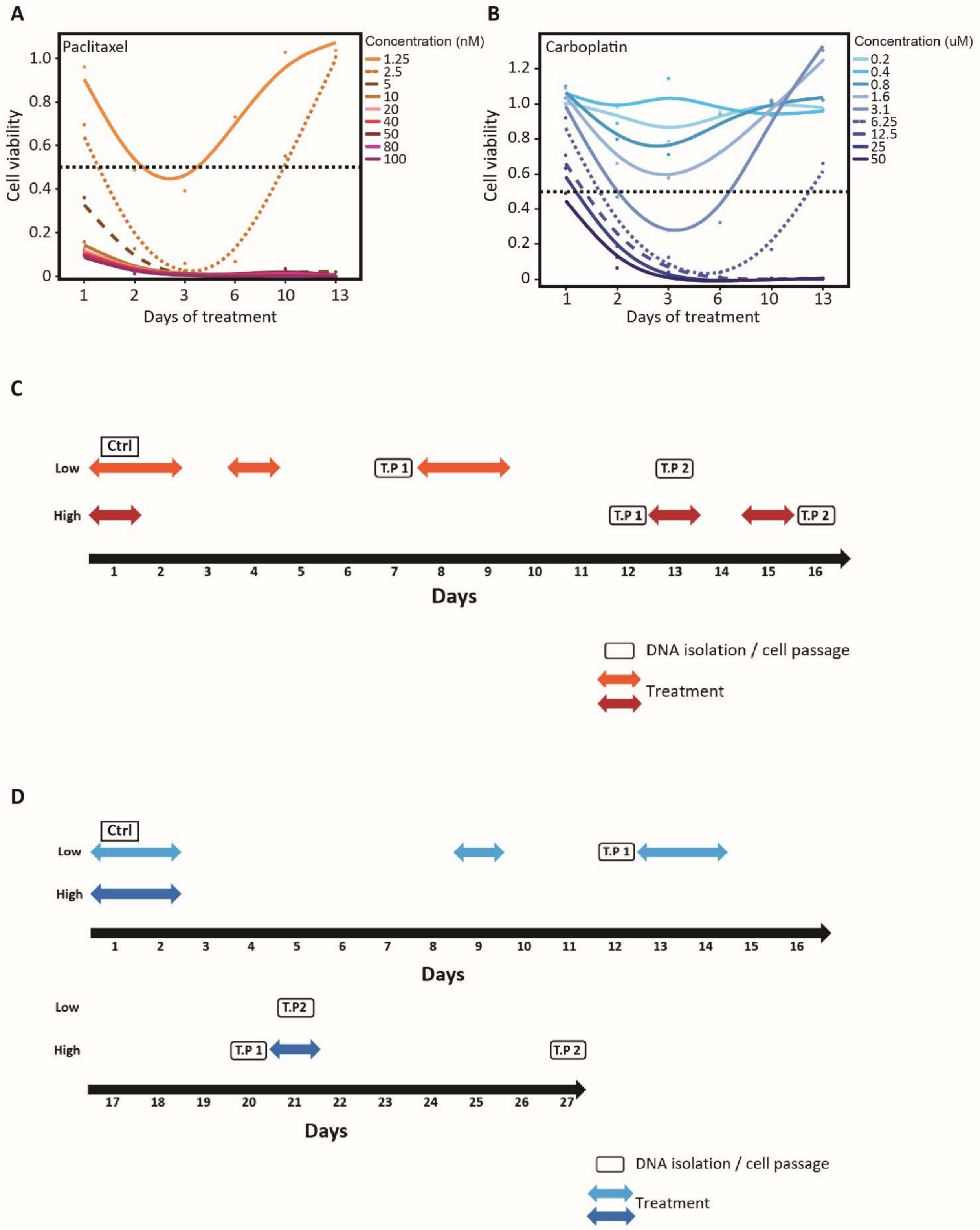
A
Paclitaxel
Cell viability
1.0
0.8
0.6
0.4
0.2
0
1
2
3
6
10
13
Days of treatment
Concentration (nM)
1.25
2.5
5
10
20
40
50
80
100
B
Carboplatin
Cell viability
1.2
1.0
0.8
0.6
0.4
0.2
0
1
2
3
6
10
13
Days of treatment
Concentration (uM)
0.2
0.4
0.8
1.6
3.1
6.25
12.5
25
50
C
Ctrl
Low
T.P 1
T.P 2
High
T.P 1
T.P 2
1 2 3 4 5 6 7 8 9 10 11 12 13 14 15 16
Days
DNA isolation / cell passage
Treatment
D
Ctrl
Low
T.P 1
High
1 2 3 4 5 6 7 8 9 10 11 12 13 14 15 16
Days
Low
T.P2
High
T.P 1
T.P 2
17 18 19 20 21 22 23 24 25 26 27
Days
DNA isolation / cell passage
Treatment

Supplementary Figure 1:

A&B. Paclitaxel (A) and carboplatin (B) calibration curves. Haploid hESCs were treated with the drugs for two weeks under conditions covering various concentrations. Cell viability was assessed using CellTiter-Glo assay. Cell death and cell recovery were monitored for optimal concentrations used for the genome-wide screens (dotted curves). X axis = days of treatment, Y axis = cell viability. **C&D.** Schematic illustration of the paclitaxel (C) and carboplatin (D) screen treatments. Black bars represent the length of each screen. Colored arrows are the treatment time point between cell death and cell recovery. Rectangles represent cell passages and library samples for DNA extraction.

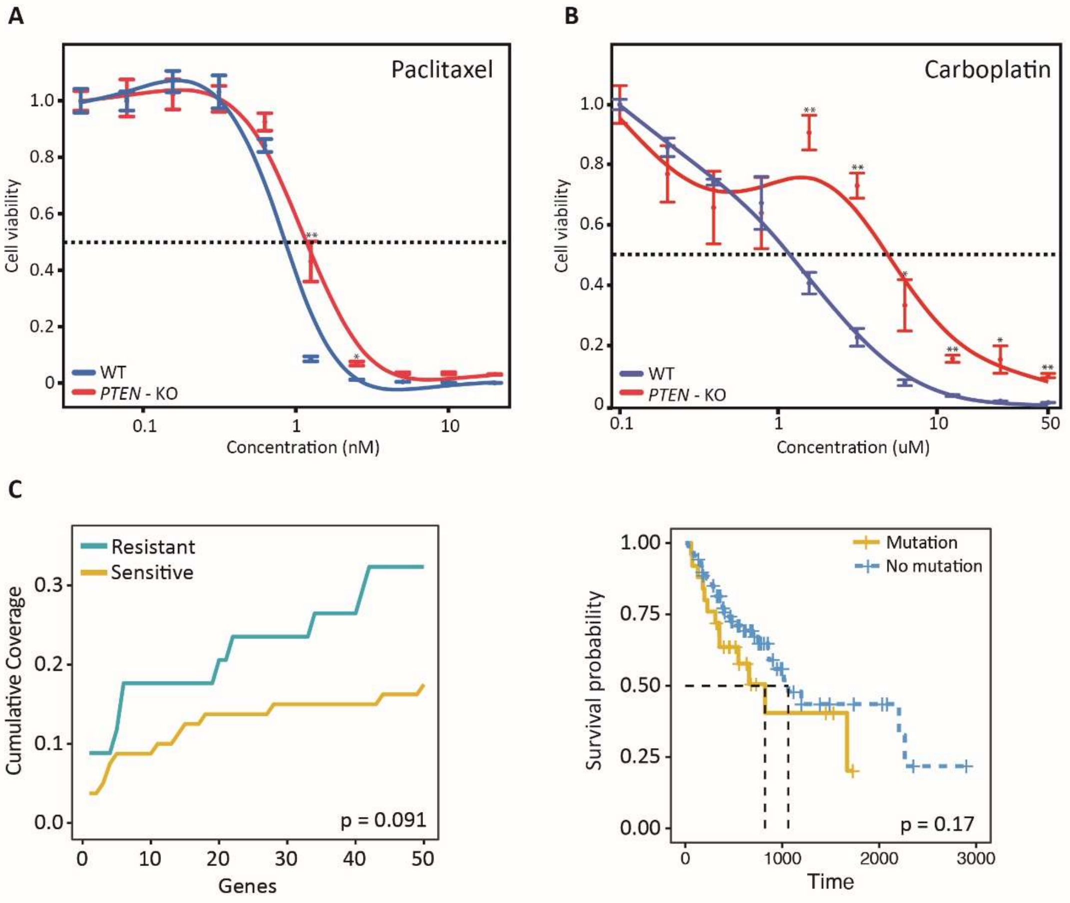

D

Paclitaxel

	0	1	2	Sum
Resistant	34	20	12	66
Sensitive	126	24	6	156
Sum	160	44	18	222

Carboplatin

	0	1	2	Sum
Resistant	68	8	9	85
Sensitive	141	9	8	158
Sum	209	17	17	243

Paclitaxel + Carboplatin

	0	1	2	Sum
Resistant	23	5	6	34
Sensitive	66	7	7	80
Sum	89	12	13	114

Supplementary Figure 2:

Response of *PTEN*-KO hESCs to paclitaxel or carboplatin treatments compared to WT cells. A&B**.** Cell viability assay of PTEN-KO and WT hESCs treated with paclitaxel (A) or carboplatin (B)**.** *PTEN*-KO cells exhibit moderate reduced sensitivity to paclitaxel treatment. **B.** *PTEN*-KO cells exhibit significant insensitivity to carboplatin treatment. * = p value < 0.05, ** = p value < 0.001

C. Left: Cumulative coverage plot of top 50 genes in paclitaxel+carboplatin. Genes are ordered by their CRISPR score. Y-axes are the cumulative coverage of patients with mutations in the genes, in the resistant and sensitive groups. Right: Kaplan Meier plots of patients with or without mutations (yellow or blue lines, respectively). **D.** Tables presenting how resistant and sensitive patients are divided across the different groups of patients that we have defined - without mutations (0), patients with mutations in genes with max CS between 1 and 5 (1), patients with mutations in genes with max CS > 5 (2). The tables presented are for paclitaxel (upper table), carboplatin (middle table) and the combination of paclitaxel and carboplatin (lower table).

Supplementary Table 1. 50 significantly enriched genes from the paclitaxel screen

Supplementary Table 2. 11 significantly enriched genes from the carboplatin screen

Supplementary Table 3. sgRNA sequences for the generation of knockout cells for *PTEN* and *TP53*.
